# An integrated approach to processing WHO-2016 verbal autopsy data: the InterVA-5 model

**DOI:** 10.1186/s12916-019-1333-6

**Published:** 2019-05-30

**Authors:** Peter Byass, Laith Hussain-Alkhateeb, Lucia D’Ambruoso, Samuel Clark, Justine Davies, Edward Fottrell, Jon Bird, Chodziwadziwa Kabudula, Stephen Tollman, Kathleen Kahn, Linus Schiöler, Max Petzold

**Affiliations:** 1 Department of Epidemiology and Global Health, Umeå University, Umeå, Sweden; 2 Institute of Applied Health Sciences, University of Aberdeen, Scotland, UK; 3 Medical Research Council/Wits University Rural Public Health and Health Transitions Research Unit (Agincourt), School of Public Health, Faculty of Health Sciences, University of the Witwatersrand, Johannesburg, South Africa; 4 Stellenbosch Institute for Advanced Study (STIAS), Wallenberg Research Centre at Stellenbosch University, Stellenbosch, South Africa; 5 Occupational and Environmental Medicine, Sahlgrenska Academy, University of Gothenburg, Gothenburg, Sweden; 6 Department of Sociology, The Ohio State University, Columbus, OH USA; 7 INDEPTH Network, Accra, Ghana; 8 Institute of Applied Health Research, University of Birmingham, Birmingham, UK; 9 Institute for Global Health, University College London, London, UK; 10 Department of Computing, University of Bristol, Bristol, UK; 11 Health Metrics, Sahlgrenska Academy, University of Gothenburg, Gothenburg, Sweden; 12 School of Public Health, Faculty of Health Sciences, University of the Witwatersrand, Johannesburg, South Africa

**Keywords:** Verbal autopsy, Mortality surveillance, Civil registration, InterVA, Cause of death, World Health Organization

## Abstract

**Background:**

Verbal autopsy is an increasingly important methodology for assigning causes to otherwise uncertified deaths, which amount to around 50% of global mortality and cause much uncertainty for health planning. The World Health Organization sets international standards for the structure of verbal autopsy interviews and for cause categories that can reasonably be derived from verbal autopsy data. In addition, computer models are needed to efficiently process large quantities of verbal autopsy interviews to assign causes of death in a standardised manner. Here, we present the InterVA-5 model, developed to align with the WHO-2016 verbal autopsy standard. This is a harmonising model that can process input data from WHO-2016, as well as earlier WHO-2012 and Tariff-2 formats, to generate standardised cause-specific mortality profiles for diverse contexts.

The software development involved building on the earlier InterVA-4 model, and the expanded knowledge base required for InterVA-5 was informed by analyses from a training dataset drawn from the Population Health Metrics Research Collaboration verbal autopsy reference dataset, as well as expert input.

**Results:**

The new model was evaluated against a test dataset of 6130 cases from the Population Health Metrics Research Collaboration and 4009 cases from the Afghanistan National Mortality Survey dataset. Both of these sources contained around three quarters of the input items from the WHO-2016, WHO-2012 and Tariff-2 formats. Cause-specific mortality fractions across all applicable WHO cause categories were compared between causes assigned in participating tertiary hospitals and InterVA-5 in the test dataset, with concordance correlation coefficients of 0.92 for children and 0.86 for adults.

The InterVA-5 model’s capacity to handle different input formats was evaluated in the Afghanistan dataset, with concordance correlation coefficients of 0.97 and 0.96 between the WHO-2016 and the WHO-2012 format for children and adults respectively, and 0.92 and 0.87 between the WHO-2016 and the Tariff-2 format respectively.

**Conclusions:**

Despite the inherent difficulties of determining “truth” in assigning cause of death, these findings suggest that the InterVA-5 model performs well and succeeds in harmonising across a range of input formats. As more primary data collected under WHO-2016 become available, it is likely that InterVA-5 will undergo minor re-versioning in the light of practical experience. The model is an important resource for measuring and evaluating cause-specific mortality globally.

## Background

The quality and performance of national health information systems varies widely around the world, correlated strongly with economic and infrastructural development. Countries that currently operate efficient and detailed health information systems, based on complete individual data, typically started from nothing 200 to 300 years ago, and began with basic registration of deaths and their causes. If the major causes of death in a population can be characterised, this leads to considerable insights in terms of health priorities and the implementation of appropriate interventions and services. However, the World Health Organization (WHO) estimated that around 50% of 56 million deaths worldwide in 2015 were not registered with information on cause [1]. Therefore, there is a great need for cost-effective, rapid and consistent tools to address this gap in the medium term.

Verbal autopsy (VA) has become an increasingly important approach for documenting deaths that otherwise pass without registration or certification, typically in lower-income countries and particularly in Africa and Asia. The basic principle of VA is that a standardised interview is conducted with family members or others having detailed knowledge of the circumstances, signs and symptoms leading to the death, and the interview data are processed into likely medical causes of death.

Necessary tools for large-scale implementation of VA comprise several essential components, which can be used in conjunction with each other to achieve the over-arching objective of making step-changes in the proportion of deaths worldwide that are appropriately registered by cause. Part of WHO’s normative global role is to develop and update standard protocols for VA interviews and cause of death reporting categories, of which the most recent version is the WHO 2016 verbal autopsy instrument (WHO-2016) [2]. This new standard, taken as a given starting point for developing InterVA-5, was primarily intended to achieve harmonisation between earlier WHO standards and the Tariff-2 system [3], which inevitably led to a larger number of interview items.

Additionally, because VA interviews typically involve multiple complex skip patterns (for example where particular interview items relate to specific age/sex groups), there are considerable efficiency gains to be made by handling VA interviews with portable data capture tools, typically implemented on smartphones or tablets. This has previously been shown to be an effective, cost-effective and acceptable approach [4]. However, the InterVA-5 software does not provide data capture functions but is designed to post-process VA interview data gathered by various means.

Although physicians have been widely used to assign individual causes of death using VA data after interviews have been conducted, that approach can be costly, slow and not always consistent between practitioners and contexts [5]. Thus, it has become more common to apply automated computerised models to VA data, which are much cheaper, faster and more consistent. It can be argued that physicians may be able to bring additional nuances to assigning causes to individual cases compared with automated models, particularly in specific research settings. Additionally, careful physician review may play a role in quality control and VA model development. Nevertheless, making any significant future impact on categorising the over 20 million uncertified deaths every year using VA will necessarily depend on using automated methods.

There are currently three families of automated VA models of relevance to the WHO-2016 standard, namely InterVA, InSilicoVA and Tariff [3, 6, 7]. Initial work on InterVA models dates from 2003 [8] and has passed through a number of iterations since. InSilicoVA built on the foundations of InterVA, aiming to achieve higher precision and measures of uncertainty by, among other things, simultaneously estimating distributions of individual cause-assignment probabilities and cause-specific mortality fractions, and differentiating between negative and unknown responses to VA responses. InSilicoVA is closely related to InterVA, using the same probability base to relate indicators and causes, and thus uses the same interview items. Tariff was first proposed in 2011 [9] and has subsequently been revised and shortened to Tariff-2, as implemented in the SmartVA-Analyze software [10].

Thus the aim of this paper is to present the development and evaluation of InterVA-5, the latest product in the InterVA family, designed to correspond to the WHO-2016 standard [11]. This builds substantially on the InterVA-4 model [6], which corresponded to the WHO 2012 verbal autopsy instrument (WHO-2012), but InterVA-5 also includes significant new concepts as well as updates based on the experience of processing hundreds of thousands of VA cases using InterVA-4. The harmonising concept behind WHO-2016 was carried forward into the design of InterVA-5, which not only directly corresponds to WHO-2016 but also incorporates backward compatibility with WHO-2012 and InterVA-4 [6], as well as coherence with Tariff-2 and the associated SmartVA-Analyze model [10]. Since WHO-2012 and Tariff-2 content are by definition separate subsets of WHO-2016, it was feasible to design InterVA-5 as a harmonising model that could handle WHO-2016, WHO-2012 or Tariff-2 datasets, in the interests of achieving wider comparability and consistency in processing existing data.

In addition, InterVA-5 incorporates a novel concept of Circumstances Of Mortality CATegories (COMCAT) as a tool that complements medical causes of death with assigning circumstantial categories to deaths, related to critical limiting factors for care seeking and utilisation processes at and around the time of death, as they occur in any specific health systems and social context. For example, for a woman whose medical cause of death is assigned as obstetric haemorrhage, her death might have occurred at home because she had no means or resources to call for help or get to a health facility; another woman with the same medical cause of death might have been inadequately managed during her delivery despite getting to a health facility. The intention of COMCAT is to make distinctions between important circumstances around a death, particularly where these may not be reflected in medical causes. The conceptual basis of COMCAT is described elsewhere [12], and a detailed operational evaluation of its implementation within InterVA-5 will follow as a separate paper.

## Implementation

The overall architecture of the InterVA-5 software follows the same general pattern as was implemented in the InterVA-4 software [6], involving the following major components:

1. System initiation—reading knowledge base and accepting user input parameters
2. Reading input data file and checking format
3. Checking data consistency, excluding errors and generating warnings
4. Processing likelihoods for each pregnancy status category, for each case
5. Processing likelihoods for each cause of death category, for each case
6. Processing likelihoods for each COMCAT, for each case
7. Post-processing output file with pregnancy status, up to three causes and COMCAT for each case

In line with the existing concept that InterVA products are made available on an open-source basis, the InterVA-5 software is issued under the GNU General Public License Version 3 (GPL3) and the accompanying knowledge base that drives the system is also freely available. In the same spirit, the specifications for the input and output files are defined in non-proprietary comma-separated variable (CSV) format. The executable software, code and full user documentation are included in the download (see linked GitHub repository) [11].

For historical reasons, the InterVA-5 software was first implemented and compiled as a run-time version in Microsoft Visual FoxPro 9.0, the same programming environment as has been used for earlier versions of InterVA. In order to co-validate the software, a parallel implementation in R was undertaken by a separate software team at another institution, and test outputs from the two separate implementations carefully checked for any discrepancies or errors. The R implementation of InterVA-5 is available via the openVA repository for open-access VA resources as open source software under GPL3 (see linked GitHub repository) [13]. The Windows and R software versions are kept synchronised and produce the same results.

All of the InterVA family of models have used a simple input format of binary questions. Up until InterVA-4, the response of interest was always defined as “yes”, even though that sometimes made the wording of questions awkward. Therefore, InterVA-5 uses a data-driven concept of a substantive response for each item, which may be “yes” (e.g. “Did (s) he have a fever?”) or “no” (e.g. “Was the placenta completely delivered?”), and the probabilistic modelling updates likelihoods for each cause category on the basis of substantive responses recorded in the VA data.

**Fig. 1:**
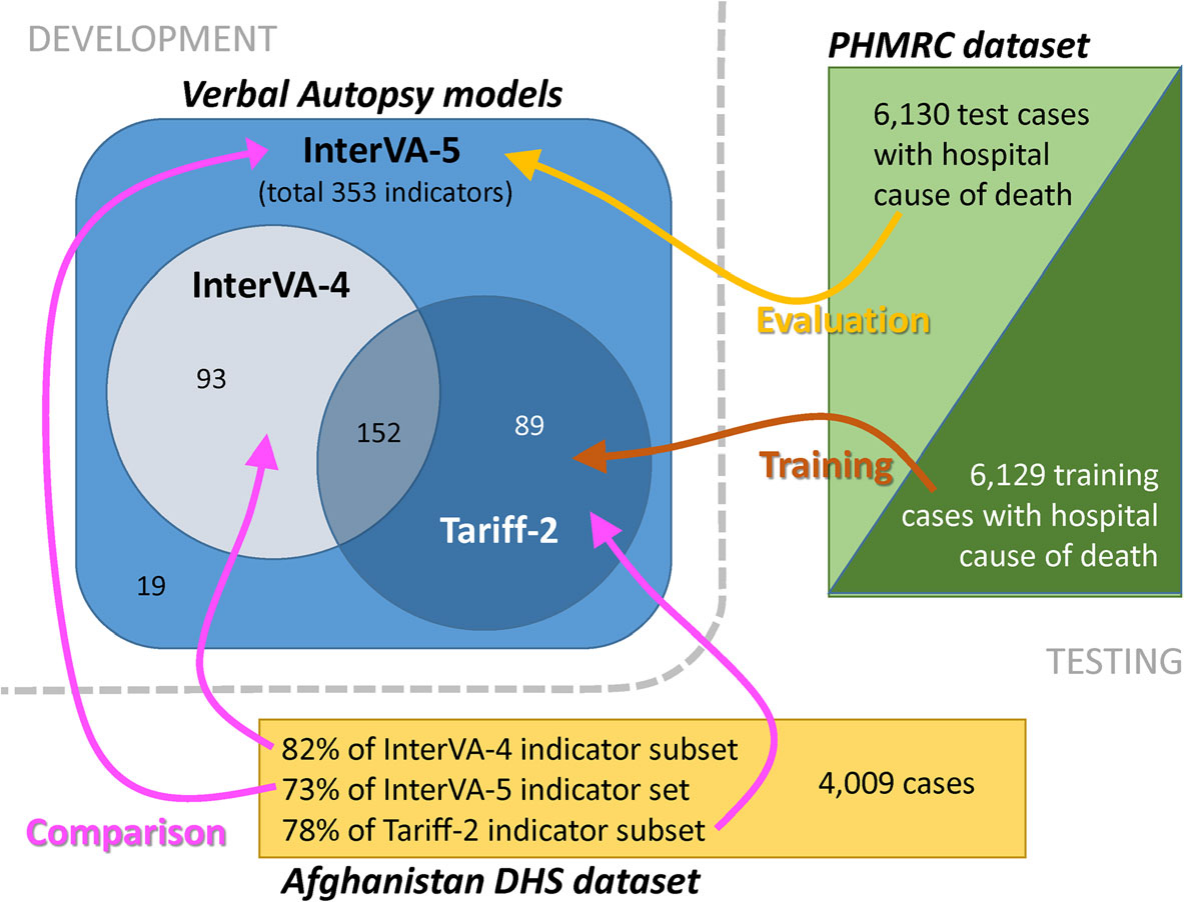
Conceptual framework for the development and testing of the InterVA-5 model

Where WHO VA items are specified in other ways (e.g. as continuous variables for duration of symptoms), InterVA takes pre-determined categories and implements each category as a binary variable. The detailed specification of WHO-2016 [14] also includes a substantial preamble of civil registration parameters which are not intended to elucidate cause of death, such as civil identity numbers and residential addresses which are not relevant to InterVA-5. Overall, the 305 items in WHO-2016 that are relevant to assigning cause of death correspond to 353 binary indicators in the InterVA-5 data input format, plus an individual identifier field. InterVA-5 input data can therefore be prepared from complete WHO-2016 data records, using a suitable script to convert to the 353 variables plus identifier required in the CSV input file. Alternatively, if there is a prior decision to use InterVA-5 as the interpretation tool, a tablet data collection tool directly designed for the InterVA-5 format can be implemented for the VA interview and the data transferred directly (for example, the MIVA utilities included in the linked GitHub repository). Since WHO-2012/InterVA-4 and Tariff-2/SmartVA-Analyze are both subsets of the WHO-2016 standard [2], it is also relatively straightforward to run conversion scripts from those data formats to the InterVA-5 input format. Figure 1 shows the combinations of input indicators for the three data formats (InterVA-5 353 indicators, InterVA-4 245 and Tariff-2 241).

The established knowledge base that drove InterVA-4 (version 4.04) was used as the basis for the InterVA-5 knowledge base. As has always been the case with the InterVA family of models, this knowledge base is an accumulated resource, based on both such data sources as are available plus syntheses of expert opinion, as previously described [15]. To move from this InterVA-4 resource to a revised version for InterVA-5, we needed to do four things:

1. Update with regard to changes in cause categories between WHO-2012 and WHO-2016
2. Update with regard to extra VA items in WHO-2016 compared with WHO-2012
3. Update according to outstanding issues reported by InterVA-4 users
4. Incorporate a knowledge base relating to the COMCAT system

The only change in mortality cause categories moving from WHO-2012 to WHO-2016 was a redefinition of the WHO-2012 category 01.11 (haemorrhagic fever) into two separate categories; 01.11 (haemorrhagic fever excluding dengue fever) and 01.12 (dengue fever). Revised probabilities for these two categories were reviewed and derived on the basis of available evidence and expert input.

The additional items in WHO-2016 compared with WHO-2012 were almost all contained in the Population Health Metrics Research Consortium (PHMRC) reference dataset [16], which was a longer precursor of the Tariff-2 format. Conditional probabilities for these items were derived by randomly selecting half of the PHMRC data as a training dataset and using that as a basis for filling the probability base for the additional items. The PHMRC reference dataset [16] was randomly divided into equal train and test datasets for revising and testing the InterVA-5 model. The training dataset was used primarily to inform conditional probability assignments in InterVA-5 for the 89 indicators (Fig. 1) present in the Tariff-2 indicator subset but not in the WHO-2012 indicator subset. The other half of the PHMRC dataset was retained as a test dataset for the new model.

A few new or revised items (e.g. the new WHO-2016 item “Did (s) he receive (or need) antiretroviral therapy (ART)?”, and splitting the InterVA-4 item “Did (s) he have fever for less than 2 weeks before death?” into “Did the fever last less than a week before death?” and “Did the fever last at least one week, but less than 2 weeks before death?”, which was specifically relevant to the additional WHO VA cause category for dengue fever) required revisions to the knowledge base on the basis of expert opinion. The complete conditional probability matrix that InterVA-5 uses is included as a spreadsheet in the download of the model [11].

A few reported issues with InterVA-4, such as implausible over-attribution of WHO, cause category 06.01 (acute abdomen) and under-attribution of 04.01 (acute cardiac), an incorrect balance between fresh and macerated stillbirths (11.01 and 11.02) and over-attribution of 01.03 (HIV/AIDS related death) in young children were addressed within the overall process of revising the knowledge base.

Social scientists contributed to a process of estimating conditional probabilities for the COMCAT factors, on the same principles as the estimation of probabilities for causes of death. This was an inherently different exercise in that no data existed in absolute terms nor indeed any sense that COMCAT outputs could be considered fundamentally correct or incorrect. This is an area that will be revisited as experience of its use grows, but the current InterVA-5 knowledge base constitutes a starting point for this novel concept.

Thus, overall the implementation of InterVA-5 constitutes a cause of death model which is fully compatible with the WHO-2016 instrument, which can also process WHO-2012 and Tariff-2 datasets, and which can assign deaths to all 64 WHO-2016 cause of death categories. The public-domain InterVA-5 model is available on an open-source basis and on a typical personal computer processes about 100 VA cases per minute.

## Results

Testing the new InterVA-5 software has been an important part of the development process. As with any software update, evaluating continuity with the previous version is important, as well as overall performance of the new version. Evaluating assignment of cause of death in any context is notoriously difficult because of a lack of any absolute comparator [17]. InterVA-4 has previously been extensively compared with the same PHMRC dataset as used here [17], physician assigned causes of death [18], co-validated with Global Burden of Disease mortality estimates [19] and deployed in large-scale mortality analyses [20]. For evaluating comparability between different approaches to modelling the same set of VA cases, the concordance correlation coefficient (CCC), as implemented in the Stata *concord* command, is a useful measure of equivalence.

Since the WHO-2016 instrument is relatively new, there are not yet any extensive VA data sources specifically collected under that protocol available for evaluation. However, some earlier VA archives do contain data including a substantial proportion of WHO-2016 items, which therefore for the present have to suffice as material for evaluating InterVA-5. There are two major objectives: firstly to compare the InterVA-5 cause of death assignments with an established, best available, reference source (even though no perfect reference source exists) and secondly to compare the performance of InterVA-5 when processing data aligned with WHO-2016, WHO-2012 and Tariff-2 input formats.

**Fig. 2:**
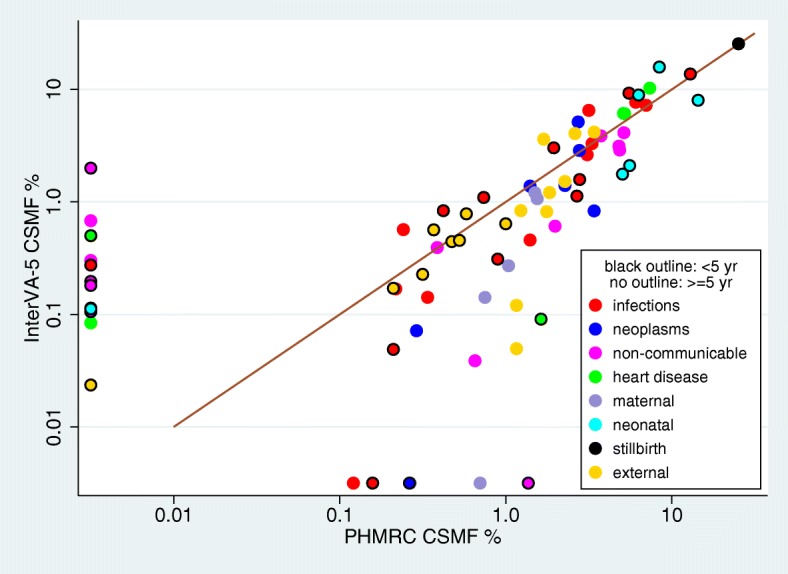
Cause-specific mortality fractions (CSMFs) by age group for 6130 deaths from the Population Health Metrics Research Consortium (PHMRC) verbal autopsy reference dataset, with PHMRC cause of death determined from clinical data at tertiary hospitals involved in final care, and processed by the InterVA-5 model from PHMRC verbal autopsy data, against the line of equivalence

Firstly, the 6130 VA records in the PHMRC test dataset were used, which covered 248/353 (70.3%) of the InterVA-5 input indicators. The strengths of the PHMRC dataset are that it includes causes of death attributed by tertiary hospitals, though not all the WHO-2016 cause of death categories are included, and its verbal autopsy data were not used as part of assigning the hospital causes of death. The PHMRC dataset causes did not differentiate between fresh and macerated stillbirths, nor between different haemorrhagic fevers, which were amalgamated into stillbirths and haemorrhagic fevers for this comparison. Because the hospital and VA processes leading to the attribution of indeterminate cause to some deaths were very different, indeterminate outcomes (1.4% for hospital and 11.1% for VA) were excluded by redistributing proportionally over all other causes for this comparison. Cause-specific mortality fractions (CSMFs) for WHO-2016 cause categories, from the hospital causes and InterVA-5, are shown in Table 1, for the 5-plus and under-5 age groups, by WHO-2016 cause categories and broad groups. InterVA-5 CSMFs were derived by aggregating individually assigned likelihoods for each cause, and dividing by total deaths. Figure 2 shows the agreement between the two sources, for deaths under 5 years and those 5 years and older, with different colours corresponding to the broad causes shown in Table 1. The points near the axes reflect rare causes that were either unrepresented or not directly comparable between the two sources, such as childhood cancers, amounting to 3.1% of the total deaths under 5 years and 1.0% of those 5 years and older. Nevertheless, we retained these points in the overall comparisons so as to take a conservative approach to assessing concordance. The CCC was 0.922 (95% CI 0.871 to 0.974) for the younger age group and 0.858 (95% CI 0.786 to 0.930) for the older age group.

**Table 1:** Cause-specific mortality fractions (CSMFs) by age group for 6130 deaths from the Population Health Metrics Research Consortium (PHMRC) verbal autopsy reference dataset, with PHMRC cause of death determined from clinical data at tertiary hospitals involved in final care, and processed by the InterVA-5 model from PHMRC verbal autopsy data. Causes of death are shown in WHO-2016 categories, as well as in broad groups

WHO-2016 cause category	CSMF % ≥ 5 years		CSMF % < 5 years	
	PHMRC	InterVA-5	PHMRC	InterVA-5
01.01 Sepsis (non-obstetric)	0.22	0.17	2.68	1.13
01.02 Acute resp infect incl pneumonia	7.00	7.23	12.93	13.72
01.03 HIV/AIDS related death	6.06	7.70	0.42	0.84
01.04 Diarrhoeal diseases	3.09	2.63	5.52	9.27
01.05 Malaria	1.40	0.46	2.79	1.58
01.06 Measles	0.12	0	0.21	0.05
01.07 Meningitis and encephalitis	0.24	0.57	1.95	3.03
01.08 & 10.05 Tetanus			0	0.11
01.09 Pulmonary tuberculosis	3.16	6.52	0.16	0.00
01.10 Pertussis			0	0.27
01.11 Haemorrhagic fever	0.34	0.14	0.89	0.31
01.99 Other and unspecified infect dis	3.31	3.30	0.74	1.10
02.01 Oral neoplasms	0.29	0.07		
02.02 Digestive neoplasms	2.73	5.14		
02.03 Respiratory neoplasms	1.40	1.38		
02.04 Breast neoplasms	2.27	1.40		
02.05 & 02.06 Reproductive neoplasms m&f	2.78	2.87		
02.99 Other and unspecified neoplasms	3.41	0.83	0.26	0.00
03.02 Severe malnutrition	0	0.30	0	2.00
03.03 Diabetes mellitus	5.14	4.12	0	0.11
04.01 Acute cardiac disease	5.12	6.13		
04.02 Stroke	7.37	10.27		
04.03 Sickle cell with crisis	0	0.08	0	0.50
04.99 Other and unspecified cardiac dis	5.19	6.10	1.63	0.09
05.01 Chronic obstructive pulmonary dis	1.98	0.61		
05.02 Asthma	0.65	0.04		
06.01 Acute abdomen	0	0.68	0	0.20
06.02 Liver cirrhosis	3.74	3.86		
07.01 Renal failure	4.81	3.13		
08.01 Epilepsy	0.39	0.39	0	0.18
09.01 Ectopic pregnancy	0	0.08		
09.02 Abortion-related death	0	1.78		
09.03 Pregnancy-induced hypertension	1.50	1.21		
09.04 Obstetric haemorrhage	1.55	1.07		
09.05 Obstructed labour	0.27	0		
09.06 Pregnancy-related sepsis	0.75	0.14		
09.07 Anaemia of pregnancy	0.70	0		
09.08 Ruptured uterus	0	0.02		
09.99 Other and unspecified maternal CoD	1.04	0.27		
10.01 Prematurity			8.41	15.81
10.02 Birth asphyxia			14.41	8.02
10.03 Neonatal pneumonia			5.05	1.77
10.04 Neonatal sepsis			5.57	2.10
10.06 Congenital malformation			6.31	8.93
10.99 Other and unspecified neonatal CoD			0	0.11
11.99 Stillbirth			25.24	25.47
12.01 Road traffic accident	3.41	4.18	0.58	0.78
12.03 Accidental fall	2.27	1.52	0.47	0.44
12.04 Accidental drowning and submersion	1.76	0.82	0.53	0.46
12.05 Accidental exposure to smoke fire & flame	1.84	1.21	1.00	0.64
12.06 Contact with venomous plant/animal	1.23	0.84	0.32	0.23
12.07 Accidental poisoning & noxious substances	1.16	0.12	0.21	0.17
12.08 Intentional self-harm	1.69	3.61		
12.09 Assault	2.61	4.06	0.37	0.56
12.99 Other and unspecified external CoD	1.16	0.05	0	0.02
98 Other and unspecified NCD	4.86	2.89	1.37	0
Broad groups				
Infections	21.63	28.72	28.29	31.41
Neoplasms	12.88	11.69	0.26	0
Cardiovascular diseases	17.68	22.58	1.63	0.59
Other non-communicable diseases	24.87	16.03	26.59	27.96
Maternal and neonatal causes	5.81	4.57	39.75	36.74
Stillbirths			25.24	25.47
External causes	17.13	16.41	3.48	3.30

**Fig. 3:**
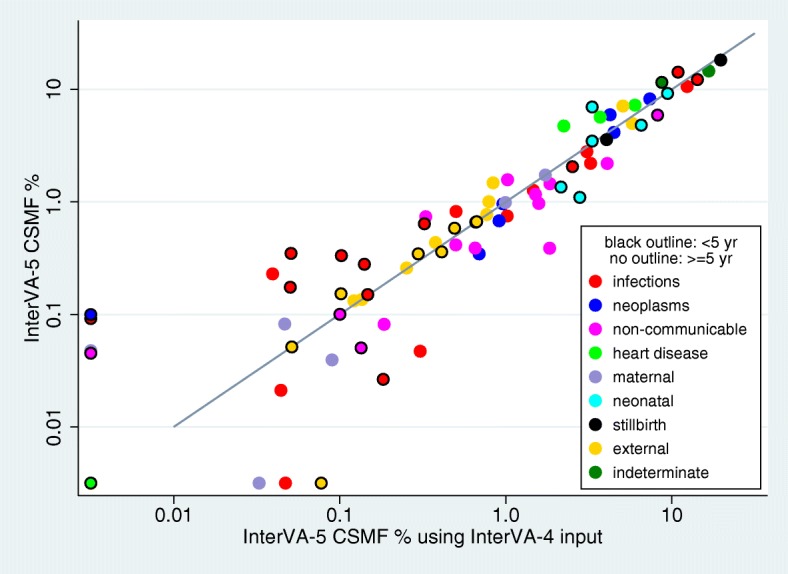
Cause-specific mortality fractions (CSMFs) by age group for 4009 deaths from the Afghanistan Mortality Survey verbal autopsy dataset, with cause of death determined by the InterVA-5 model using WHO-2016 and WHO-2012 input datasets, against the line of equivalence

**Fig. 4:**
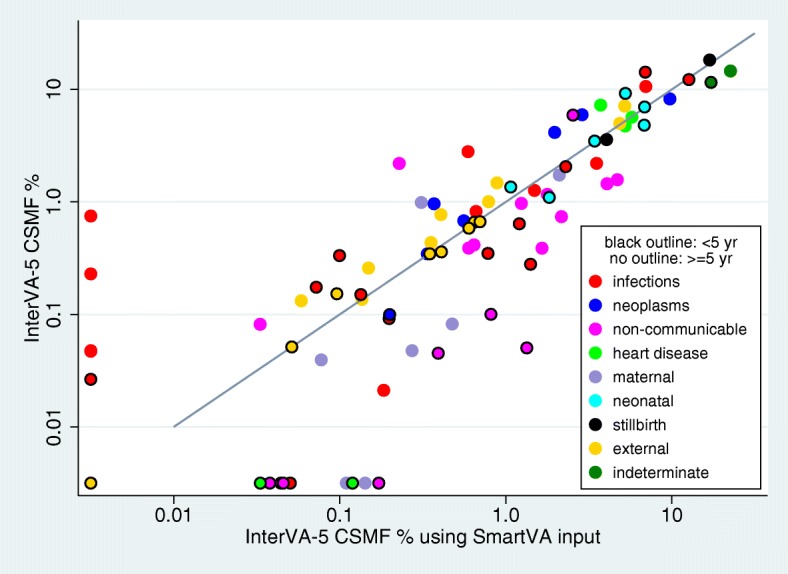
Cause-specific mortality fractions (CSMFs) by age group for 4009 deaths from the Afghanistan Mortality Survey verbal autopsy dataset, with cause of death determined by the InterVA-5 model using WHO-2016 and Tariff-2 input datasets, against the line of equivalence

For the second objective of testing the performance of the new InterVA-5 software when confronted by different subsets of input indicators, the Afghanistan 2010 national mortality survey dataset [21] was used, being a national all-age population-based dataset that was collected independently of any of the WHO-2016, WHO-2012 or Tariff-2 protocols, but included 257/353 (72.8%) of the InterVA-5 items. When reduced to the InterVA-4 and Tariff-2 subsets of the InterVA-5 items, 202/245 (82.4%) and 188/241 (78.0%) respectively of those subsets were available, as shown in Fig. 1. Table 2 shows the InterVA-5 outputs for the three datasets based on the WHO-2016, WHO-2012 and Tariff-2 standards for the under-5 and 5-plus age groups, by WHO-2016 cause categories and broad groups. Figure 3 shows the agreement between the outputs using the InterVA-5 and InterVA-4 datasets, and Fig. 4 the InterVA-5 and Tariff-2 datasets. CCCs for InterVA-4 were 0.968 (95% CI 0.947 to 0.988) for the under-5 age group, and 0.961 (95% CI 0.940 to 0.983) for the 5-plus age group; for Tariff-2, the CCCs were 0.918 (95% CI 0.869 to 0.968) for the under-5 age group and 0.871 (95% CI 0.806 to 0.936) for the 5-plus age group. Points near the axes in these comparisons reflect very rare causes that were barely measurable from this dataset.

**Table 2:** Cause-specific mortality fractions (CSMFs) by age group for 4009 deaths from the Afghanistan Mortality Survey verbal autopsy dataset, with cause of death determined by the InterVA-5 model using datasets extracted on the basis of WHO-2016, WHO-2012 and Tariff-2 indicator formats. Causes of death are shown in WHO-2016 categories, as well as in broad groups

WHO-2016 cause category	CSMF % ≥ 5 years			CSMF % < 5 years		
	WHO-2016	WHO-2012	Tariff-2	WHO-2016	WHO-2012	Tariff-2
01.01 Sepsis (non-obstetric)	0.02	0.04	0.18	0.64	0.32	1.21
01.02 Acute resp infect incl pneumonia	2.20	3.24	3.52	12.24	14.27	12.68
01.03 HIV/AIDS related death	0.75	1.02	0	0.33	0.10	0.10
01.04 Diarrhoeal diseases	2.80	3.08	0.59	14.25	10.91	6.92
01.05 Malaria	0.05	0.30	0	0.03	0.18	0
01.06 Measles	0.05	0	0	0.17	0.05	0.07
01.07 Meningitis and encephalitis	1.26	1.46	1.49	2.06	2.52	2.30
01.08 Tetanus	0	0.05	0			
01.09 Pulmonary tuberculosis	10.62	12.37	6.97	0.15	0.15	0.13
01.10 Pertussis				0.35	0.05	0.78
01.11 Haemorrhagic fever (non-dengue)	0.23	0.04	0	0	0	0.05
01.12 Dengue fever				0.09	0	0.20
01.99 Other and unspecified infectious disease	0.82	0.50	0.66	0.28	0.14	1.41
02.01 Oral neoplasms	0.35	0.69	0.34			
02.02 Digestive neoplasms	8.24	7.37	9.74			
02.03 Respiratory neoplasms	4.15	4.49	1.97			
02.04 Breast neoplasms	0.96	0.96	0.37			
02.05 & 02.06 Reproductive neoplasms m&f	0.68	0.91	0.56			
02.99 Other and unspecified neoplasms	5.96	4.25	2.88	0.10	0	0.20
03.01 Severe anaemia	0.08	0.18	0.03	0	0	0.04
03.02 Severe malnutrition	2.20	4.08	0.23	5.92	8.21	2.54
03.03 Diabetes mellitus	1.45	1.85	4.07	0.05	0	0.39
04.01 Acute cardiac disease	4.73	2.24	5.24			
04.02 Stroke	7.25	6.00	3.73	0	0	0.03
04.99 Other and unspecified cardiac disease	5.67	3.71	5.75	0	0	0.12
05.01 Chronic obstructive pulmonary disease	0.97	1.58	1.24			
05.02 Asthma	0.39	0.65	0.60	0	0	0.05
06.01 Acute abdomen	0.39	1.84	1.65	0.05	0.13	1.34
06.02 Liver cirrhosis	1.58	1.02	4.70	0	0.09	2.12
07.01 Renal failure	0.74	0.33	2.16	0	0	0.17
08.01 Epilepsy	0.41	0.50	0.64	0.10	0.10	0.81
09.02 Abortion-related death	0.05	0	0.27			
09.03 Pregnancy-induced hypertension	0.99	0.99	0.31			
09.04 Obstetric haemorrhage	1.73	1.73	2.10			
09.05 Obstructed labour	0	0.03	0			
09.06 Pregnancy-related sepsis	0.04	0.09	0.08			
09.07 Anaemia of pregnancy	0	0	0.11			
09.08 Ruptured uterus	0.08	0.05	0.48			
09.99 Other and unspecified maternal cause	0	0	0.14			
10.01 Prematurity				6.99	3.31	6.85
10.02 Birth asphyxia				9.22	9.43	5.25
10.03 Neonatal pneumonia				4.81	6.55	6.82
10.04 Neonatal sepsis				1.36	2.15	1.07
10.06 Congenital malformation				3.48	3.31	3.43
10.99 Other and unspecified neonatal cause				1.10	2.79	1.83
11.01 Fresh stillbirth				18.27	19.71	16.91
11.02 Macerated stillbirth				3.58	4.05	4.05
12.01 Road traffic accident	7.13	5.07	5.21	0.66	0.66	0.65
12.03 Accidental fall	1.48	0.84	0.88	0.58	0.49	0.60
12.04 Accidental drowning and submersion	1.01	0.79	0.79	0.67	0.67	0.70
12.05 Accidental exposure to smoke fire & flame	0.44	0.38	0.35	0.35	0.30	0.35
12.06 Contact with venomous plant/animal	0.14	0.14	0.14	0.36	0.41	0.41
12.07 Accidental poisoning & noxious substances	0.13	0.12	0.06	0	0.08	0
12.08 Intentional self-harm	0.77	0.77	0.41			
12.09 Assault	4.99	5.78	4.86	0.05	0.05	0.05
12.99 Other and unspecified external cause	0.26	0.25	0.15	0.15	0.10	0.10
98 Other and unspecified NCD	1.17	1.51	1.78	0	0	0.04
99 Indeterminate	14.60	16.70	22.57	11.56	8.71	17.23
Broad groups						
Infections	18.80	22.10	13.41	30.59	28.69	25.85
Neoplasms	20.34	18.67	15.86	0.10	0	0.20
Cardiovascular diseases	17.65	11.95	14.72	0	0	0.15
Other non-communicable diseases	9.37	13.55	17.10	6.12	8.54	7.50
Maternal and neonatal causes	2.89	2.89	3.49	26.96	27.54	25.25
Stillbirths				21.85	23.76	20.96
External causes	16.35	14.14	12.85	2.82	2.76	2.86
Indeterminate	14.60	16.70	22.57	11.56	8.71	17.23

**Fig. 5:**
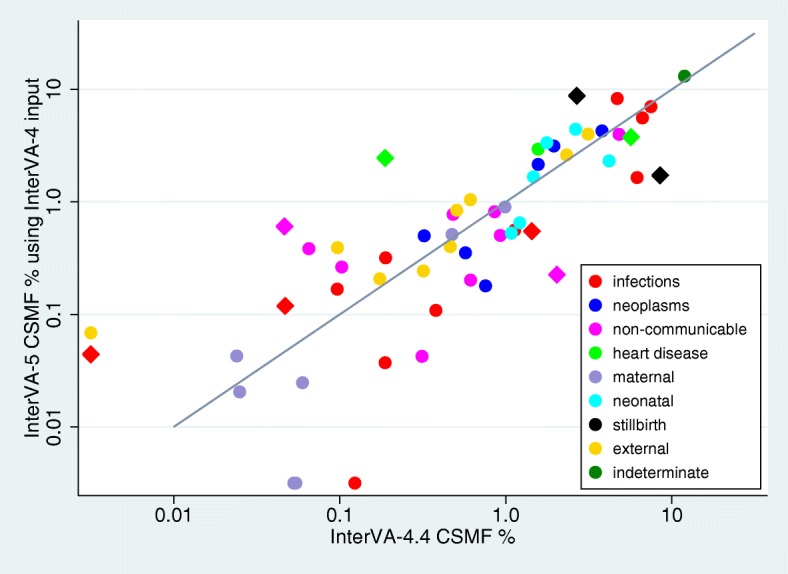
Cause-specific mortality fractions (CSMFs) by age group for 4009 deaths from the Afghanistan Mortality Survey verbal autopsy dataset, with cause of death determined by the InterVA-5 and InterVA-4 (version 4.04) model using WHO-2012 input datasets, against the line of equivalence. Diamond-shaped markers represent causes intentionally revised in the InterVA-5 model

Finally, as with any software update, it is important to demonstrate version continuity together with the effects of intentional changes as part of the update process. Figure 5 shows the Afghanistan dataset as processed by InterVA-4 (version 4.04), compared with the new InterVA-5 software processing the InterVA-4 subset of inputs. Excluding the intentional changes (shown as diamond-shaped markers in Fig. 5), CCC was 0.909 (95% CI 0.860 to 0.958).

## Discussion

The development of the InterVA-5 model follows our established practice of providing analytical models for verbal autopsy data that correspond to international WHO VA standards. WHO-2016 was specifically developed as a harmonisation of various existing VA standards, and accordingly InterVA-5 was specifically developed to be, as far as technically possible, a unifying and updated model capable of handling a range of input formats corresponding to various VA standards. One might expect that InterVA-5 would perform most robustly when used with data meeting the full WHO-2016 specification, therefore having the maximum amount of information available. However, it is important, as demonstrated here, that it can also perform reasonably comparably with WHO-2012 and Tariff-2 input formats, even though those do not fully meet current standards. Tracking mortality patterns consistently over time and place is critical in terms of evaluating health and development policy and therefore the ability to process earlier VA data collected under previous standards is strategically important.

The absolute accuracy of VA in general, and in assessing specific models for assigning cause of death from VA data, raises difficult questions which have been extensively explored in various settings. In many ways, the performance of VA methods has received more scientific scrutiny than the sometimes serendipitous nature of individual physicians’ certification of deaths. There is no process for cause of death attribution leads to absolute “truth” for every case, and the lack of precise comparators often makes assessments of various VA methods contentious. Here we have made use of the interesting, though by no means perfect, PHMRC reference dataset [16]. This at least provides cause of death as clinically assigned by the tertiary facilities in which the deaths occurred, which was backed up by laboratory and diagnostic evidence. Nevertheless, one can find cases where correspondence between the clinical cause of death and responses to questions in the VA interview was not obviously congruent. However, as evident in Fig. 2, the overall similar patterns of mortality between InterVA-5 and the PHMRC data, albeit in a tertiary hospital population unrepresentative of more usual VA applications, are an encouraging starting point. The comparison of broad cause categories presented at the end of Table 1 also suggests that at an overall level there are not major differences that would give rise to public health concerns.

Earlier versions of InterVA models have been used extensively and have been seen to deliver largely plausible findings over a wide range of settings and mortality patterns [20]. Nevertheless, as with any modelling exercise, there are always possibilities for improvement, with the caveat that a so-called improvement in one respect must not lead to deterioration in other respects. Our detailed evaluations reported here, using the Afghan VA dataset, of the new InterVA-5 model in relation to its antecedents are therefore very important. Although it may be difficult to compare performance on very rare causes of death, Figs. 3, 4 and 5 clearly demonstrate that on a population basis there is strong overall consistency between InterVA-5 and earlier models and standards. Demonstrating this continuity between models is important for long-term studies of population mortality.

As yet, very few primary data have been collected under the WHO-2016 standard, which limits the field applications of InterVA-5 to date, and hence the source material for evaluating InterVA-5. As was the case with InterVA-4, which underwent a series of minor modifications in response to feedback, issued as new versions of the public software over the past 5 years, it is anticipated that InterVA-5 will experience a similar software life cycle as experience of its use extends. We therefore particularly welcome feedback from InterVA-5 users.

## Conclusions

At present, InterVA-5 and the related InSilico model are the only tools for analysing VA data which are fully compatible with the WHO-2016 standard (in terms of VA interview input items and deriving all of the WHO-VA cause of death categories as outputs). The InterVA-5 model brings the additional advantage of being able to handle data from the earlier WHO-2012 and Tariff-2 standards reasonably well, thus bringing a helpful degree of harmonisation across the interpretation of various VA data formats. This harmonisation is important for monitoring long-term trends over periods when different VA standards have been used. As with any VA model, the usefulness of the outputs depends on using good quality source material from VA interviews, carefully preparing input data, and appropriately processing and interpreting outputs. It is likely that widespread use of the model will lead to future minor refinements. The free availability of InterVA-5 means that large quantities of VA data, even into the millions of cases which could be generated in national civil registration processes, can now be processed cheaply, feasibly and consistently. Current measurement needs for the United Nations’ Sustainable Development Goals, as well as monitoring and evaluating progress towards WHO’s visions for Universal Health Care and non-communicable disease control, make standardised cause-specific mortality measurement techniques, as implemented in InterVA-5, an essential part of the global toolkit [22]. In addition, InterVA-5 is a tool that can readily be used by national or regional health services to track local mortality patterns.

## Availability and requirements

Project name: InterVA-5

Project home page: www.interva.net

Operating system(s): runs in a DOS window on a personal computer; platform independent

Programming language: FoxPro (compiled into a runtime format)

Other requirements: runs directly from the folder into which it is downloaded

Licence: GNU General Public Licence Version 3

Any restrictions to use by non-academics: none

## Abbreviations

CCC: Concordance correlation coefficient
COMCAT: Circumstances Of Mortality CATegories
CSMF: Cause-specific mortality fraction
CSV: Comma-separated variable
PHMRC: Population Health Metrics Research Consortium
VA: Verbal autopsy
WHO: World Health Organization
WHO-2012: World Health Organization 2012 verbal autopsy standard
WHO-2016: World Health Organization 2016 verbal autopsy standard
